# Middle Cerebral Artery Doppler and Cerebroplacental Ratio Predict Adverse Neurological Outcomes in Fetal Growth Restriction

**DOI:** 10.1002/pd.70189

**Published:** 2026-05-28

**Authors:** Silvia Visentin, Ambrogio Pietro Londero, Giulia Lo Storto, Eugenio Ragazzi, Sabrina Salvadori, Eugenio Baraldi, Erich Cosmi

**Affiliations:** ^1^ Padova Hospital and Department of Women's and Children's School of Medicine Padova Hospital and University Padova Italy; ^2^ Department of Neuroscience, Rehabilitation, Ophthalmology, Genetics Maternal and Infant Health University of Genova Genova Italy; ^3^ Obstetrics and Gynecology Unit IRCCS Istituto Giannina Gaslini Genova Italy; ^4^ Department of Pharmaceutical and Pharmacological Sciences University of Padova Padova Italy

**Keywords:** cerebroplacental ratio (CPR), fetal growth restriction (FGR), head circumference (HC), middle cerebral artery (MCA) Doppler, neurological outcomes, prenatal care

## Abstract

**Objective:**

To assess the efficacy of middle cerebral artery (MCA) Doppler ultrasonography in predicting neurological and respiratory outcomes in fetuses with fetal growth restriction (FGR) and to establish a correlation between MCA Doppler and head circumference (HC).

**Method:**

Retrospective cohort study examining singleton pregnancies affected by FGR (2018–2019). MCA and Cerebroplacental Ratio (CPR) Doppler indices (*z*‐scores) alongside neonatal neurological and respiratory outcomes were gathered.

**Results:**

MCA (OR 0.51, 95% CI 0.32–0.82) and CPR (OR 1.36, 95% CI 1.10–1.68) were identified as significant predictors of neurological outcomes (*p* < 0.05). Gestational age and CPR exhibited comparable univariate accuracy; however, the multivariate model demonstrated superior performance, achieving an area under the curve of 80.30%. Correlation analysis indicated a significant relationship between neonatal HC and MCA PI *z*‐score in univariate analysis, but this was not confirmed in multivariate analysis.

**Conclusion:**

MCA Doppler and CPR are effective predictors of adverse neurological outcomes in FGR, potentially aiding risk stratification in clinical practice.

## Key Points

- What is already known?
  ◦ Fetal growth restriction is linked to adverse neurodevelopment; MCA Doppler and CPR reflect fetal hemodynamic adaptation, but prognostic performance for neurological morbidity remains variable.
- What does this study add?
  ◦ In an FGR cohort, MCA PI *z*‐score and CPR *z*‐score predicted composite adverse neurological outcomes independent of gestational age, improving risk stratification.

**Abbreviations**
AUC: Area Under the Curve
CPR: Cerebroplacental Ratio
FGR: Fetal Growth Restriction
GDM: Gestational Diabetes Mellitus
HC: Head Circumference
HDP: Hypertensive Disorders of Pregnancy
MCA: Middle Cerebral Artery
MRI: Magnetic Resonance Imaging
OGTT: Oral Glucose Tolerance Test
OR: Odds Ratio
PI: Pulsatility Index
PPHN: Persistent Pulmonary Hypertension of the Newborn
RDS: Respiratory Distress Syndrome
ROC: Receiver Operating Characteristic
UA: Umbilical Artery
WHO: World Health Organization

## 1 Introduction

Fetal growth restriction (FGR) affects around 10% of pregnancies and poses significant risks for adverse neurological and developmental outcomes [1, 2]. Early prediction of these outcomes is crucial for timely and targeted interventions to mitigate potential long‐term impairments. Despite advancements in perinatal care, predicting neurological outcomes in FGR fetuses remains challenging due to the complex interplay of factors affecting fetal development. This challenge underscores the need for reliable diagnostic tools that can be used during pregnancy to accurately predict and address potential developmental issues.

Head circumference (HC) measurement is a significant indicator of neurological outcomes in premature infants, underscoring its importance in early evaluations to guide interventions and improve developmental predictions [1]. Genetic factors contribute to this correlation, with Vogelezang et al. (2022) identifying associations between early‐life HC, adult intracranial volume, and cognitive outcomes [3]. HC is also valuable for monitoring infants and children who have experienced brain injuries. Leong et al. (2021) found that children with perinatal stroke exhibited a notable decrease in head growth compared to their peers, which was linked to poorer outcomes, highlighting its prognostic value [4].

Morgan et al. (2014) emphasize the significant role of HC in predicting neurodevelopmental outcomes in premature infants and the necessity of nutritional interventions for optimal postnatal head growth [5]. Although their study focuses on postnatal development, it indirectly underscores the importance of prenatal Doppler assessments, particularly middle cerebral artery (MCA) measurements, in predicting long‐term outcomes. Incorporating MCA Doppler assessments into regular prenatal care can enhance the early detection of infants at risk of developmental delays, enabling timely and specific interventions [5].

Currently, there is a knowledge gap regarding the specific predictors of neurological outcomes in fetuses with FGR. Traditional indicators, such as clinical observations and Apgar scores, have limited predictive efficacy, often failing to comprehensively evaluate future neurodevelopmental trajectories [1]. Addressing this gap is essential for improving patient care and providing valuable insights for clinical practices and resource allocation in perinatal health. Early prediction of potential neurodevelopmental disabilities can inform strategies to support optimal brain development, thereby reducing the risk of cognitive impairments and improving overall outcomes.

The primary objective of this study was to evaluate the effectiveness of MCA Doppler ultrasonography as a predictive tool for assessing neurological outcomes in FGR fetuses. Secondary aims were to evaluate the accuracy of MCA Doppler measurements in predicting adverse newborn respiratory outcomes and correlate MCA Doppler with HC.

## 2 Material and Methods

### 2.1 Design

This retrospective cohort study was conducted through a clinical chart review to assess the effectiveness of MCA Doppler ultrasonography in predicting neurological outcomes in FGR fetuses. Ethical approval was obtained from the ethics committee of the University of Padua, and routine informed consent was secured from all participants as part of their standard clinical care.

### 2.2 Setting and Sample

The study was carried out at the Clinic of Obstetrics and Gynecology, University of Padua, between 2018 and 2019. This setting provided a consistent and controlled environment for data collection, ensuring the reliability and validity of the study findings. The target population included singleton pregnancies followed in a high‐risk clinic due to suspected FGR. Inclusion criteria were singleton pregnancies with a pre‐pregnancy diagnosis of FGR. Exclusion criteria included multiple pregnancies, non‐FGR fetuses, no ultrasound data within 1 week before birth, and known genetic syndromes.

### 2.3 Data Collection and Measurement

Data were collected by trained obstetricians and gynecologists specializing in maternal‐fetal medicine. Information was extracted from clinical charts, including routine MCA Doppler assessments, neurological and respiratory outcomes, and relevant perinatal data. Data collection was retrospectively performed.

FGR was defined based on the consensus established by Gordijn et al. (2016) through a Delphi procedure [6]. This definition encompasses both early (< 32 weeks) and late (≥ 32 weeks) FGR, taking into account parameters such as abdominal circumference, estimated fetal weight, and Doppler measurements of the umbilical and uterine arteries and cerebroplacental ratio [6, 7]. In this study, hypertensive disorders of pregnancy (HDP) were defined using the International Society for the Study of Hypertension in Pregnancy (ISSHP) guidelines [8]. Gestational diabetes mellitus (GDM) was diagnosed using a two‐hour oral glucose tolerance test (OGTT) with 75 g of glucose, performed between the 16–18th and between the 24–28th weeks of gestation according to predetermined risk factors [9]. GDM was defined based on the following thresholds: fasting glucose ≥ 5.1 mmol/L, one‐hour glucose ≥ 10.0 mmol/L, or two‐hour glucose ≥ 8.5 mmol/L [9].

The primary variables measured were composite negative neurological outcomes and composite adverse respiratory outcomes. The composite negative neurological outcome included pathologic findings at brain ultrasound, pathologic findings at brain magnetic resonance imaging (MRI), intraventricular hemorrhage, periventricular leukomalacia, altered neurological examination, neuromotor problems, or pathological neuropsychiatric assessment. The composite negative respiratory outcome included invasive mechanical ventilation, hyaline membrane disease, apnea of prematurity, use of surfactant, pulmonary hypertension, bronchopulmonary dysplasia, and pulmonary hemorrhage. Perinatal death was also documented as a critical outcome variable. Diagnostic criteria for each component of the composite outcomes are detailed below.

Brain ultrasound and MRI were performed as part of the routine management procedure [10]. Any related injury pattern was documented as a pathologic finding [10]. In particular, intraventricular hemorrhage was diagnosed and classified using Papile's definitions [11]. The MRI results, obtained as part of the standard procedure outlined in Volpe's study, confirmed the diagnosis of periventricular leukomalacia [12]. Respiratory distress syndrome (RDS) (hyaline membrane disease) was diagnosed based on the need for mechanical ventilation and oxygen for at least 48 h, as well as the presence of specific radiographic chest findings such as air bronchograms and a reticulogranular appearance of the pulmonary parenchyma [13]. Bronchopulmonary dysplasia was defined using Walsh's definitions from their 2003 publication [14]. Apnea of prematurity is defined as a disorder characterized by apneic episodes lasting ≥ 20 s, episodes accompanied by a heart rate ≤ 70 beats per minute, and oxygen saturation (SpO2) < 80%. Echocardiography was used to diagnose persistent pulmonary hypertension in the newborn (PPHN) [15, 16]. Echocardiography was performed on any infant who presented with unremitting cyanosis unrelated to parenchymal lung disease to rule out structural heart defects and confirm the presence of PPHN. Pulmonary hemorrhage in newborns was defined as the discharge of large amounts of bloody fluid from the endotracheal tube or respiratory tract in critically ill newborns experiencing acute clinical deterioration, such as respiratory decompensation necessitating intubation or additional support [17]. Traces of blood in suctioned respiratory secretions did not indicate pulmonary hemorrhage [17]. An altered neurological examination was defined as an infant exhibiting abnormalities in standard neurological assessments, such as abnormal muscle tone, reflexes, or movements that suggested potential neurological dysfunction. In the case of follow‐up visits, neuromotor problems were defined as difficulties or abnormalities in the control and coordination of muscle movements, such as delayed motor milestones, abnormal posturing, or persistent primitive reflexes beyond the expected age. Pathological neuropsychiatric assessment was determined during follow‐up visits when an infant demonstrated significant deviations in neuropsychiatric evaluations, such as cognitive, behavioral, or emotional disturbances, as assessed by a pediatric neuropsychiatrist. Neonatal death was defined as any event that occurred in the first 28 days or during postpartum hospitalization [18]. Clinical and instrumental assessments were conducted as part of standard clinical care during postpartum hospitalization and prior to or at discharge. Neurological and neuropsychiatric evaluations were performed during outpatient visits at the 12‐month follow‐up.

The estimated fetal weight (EFW) was calculated using the Hadlock formula, as previously described [19, 20]. The EFW *z*‐score was then calculated using the method described by Hadlock et al. (1991) [20, 21]. The INTERGROWTH‐21st standards were used to compute birth weight, length, and HC *z*‐scores [22, 23]. The World Health Organization (WHO) growth standards were used to calculate infant weight, length, and HC *z*‐scores [24, 25]. Fetal Doppler *z*‐scores, including those for the umbilical artery (UA), MCA, and cerebroplacental ratio (CPR), were calculated using the reference ranges and methods described by Ebbing et al. (2007) in their study on longitudinal reference ranges for fetal MCA blood flow velocities and pulsatility index (PI), as well as the CPR [26].

### 2.4 Data Analysis

Data analysis was conducted using R software (version 4.4.0). The normality of the data distribution was assessed using the Kolmogorov‐Smirnov test. Univariate and multivariate logistic regressions were performed to evaluate the association between MCA Doppler measurements and adverse neurological and respiratory outcomes. Similarly, univariate and multivariate linear regressions were used to assess the relationship between MCA Doppler values and postnatal HC growth. Each multivariable logistic regression model included a single predictor of interest adjusted for two pre‐specified covariates (gestational age at delivery and birthweight *z*‐score). Missing data in covariates were addressed using multiple imputation with five imputed datasets for multivariable analyses only (imputed values accounted for < 2% of the data); univariable analyses were performed using available data without imputation. Model performance was evaluated using the receiver operating characteristic (ROC) area under the curve (AUC). Differences between AUCs were assessed using the DeLong test. Statistical significance was set at *p* < 0.05. The analysis included confidence intervals (95%) to evaluate the precision of the estimates. The Pearson correlation test was used to create the correlation matrix, and the false discovery rate was applied to the *p*‐values.

## 3 Results

During the study, 98 singleton pregnancies were monitored for suspected FGR. A total of 83 cases were included in the analysis, with 15 excluded due to a lack of ultrasound examination data within one week before birth.

The average maternal age was 33.49 (± 5.7) years. 39.76% of the pregnancies were complicated by hypertensive disorders of pregnancy (HDP), while 8.43% had gestational diabetes mellitus (GDM) (Table 1). All deliveries were performed by cesarean section, with RDS prophylaxis given to all cases delivered before 34 weeks of gestation. Details of the study population, ultrasound examination, and outcomes are presented in Table 1.

**TABLE 1:** Population description.

Pregnancy characteristics	
Mother age (years)	33.49 (± 5.7)
HDP	39.76% (33/83)
GDM	8.43% (7/83)
Ultrasound examination before delivery	
EFW (grams)	1178.53 (± 452.49)
EFW (*z*‐score)	−2.96 (± 0.94)
MCA PI	1.5 (± 0.31)
MCA PI (*z*‐score)	−1.99 (± 1.14)
UA PI	1.62 (± 0.57)
UA PI (*z*‐score)	3.52 (± 2.44)
CPR	1.07 (± 0.52)
CPR (*z*‐score)	−3.51 (± 1.72)
Birth and newborn data	
RDS prophylaxis	85.54% (71/83)
Gestational age (weeks)	31.38 (± 2.94)
Neonatal sex	
Male	49.4% (41/83)
Female	50.6% (42/83)
Birth weight (grams)	1190.65 (± 446.07)
Birth weight (*z*‐score)	−1.73 (± 0.93)
Birth length (grams)	37.43 (± 4.41)
Birth length (*z*‐score)	−1.65 (± 1.21)
Birth HC (cm)	27.39 (± 2.89)
Birth HC (*z*‐score)	−0.95 (± 1.03)
Apgar score (1st min)	7 (6|8)
Apgar score (5th min)	8 (8|9)
Apgar score (10th min)	9 (8|9)
Cord blood pH	7.3 (± 0.08)
Cord blood BE	−2.55 (± 2.44)
Composite neurological outcome	
Pathologic finding at brain ultrasound	39.76% (33/83)
Pathologic finding at brain MRI	2.41% (2/83)
Intraventricular hemorrhage	4.94% (4/81)
Periventricular leukomalacia	0% (0/81)
Altered neurological examination	4.94% (4/81)
Neuromotor problems	3.75% (3/80)
Pathological neuropsychiatric assessment	0% (0/83)
Composite neurological outcome	45.78% (38/83)
Composite respiratory outcome	
Invasive mechanical ventilation	37.04% (30/81)
Hyaline membrane disease	60.49% (49/81)
Apnea of prematurity	61.73% (50/81)
Use of surfactatnt	40.74% (33/81)
Pulmonary hypertension	3.7% (3/81)
Bronchopulmonary dysplasia	11.11% (9/81)
Pulmonary hemorrhage	2.47% (2/81)
Composite respiratory outcome	80.72% (67/83)
Infant discharged from hospital	
Hospitalization length (days)	40.1 (± 36.28)
Neonatal death	2.41% (2/83)
Infant weight at discharge (grams)	1848.4 (± 496.92)
Infant weight at discharge (*z*‐score)	−5.44 (± 1.16)
Infant length (grams)	42.6 (± 4.46)
Infant length (*z*‐score)	−6.61 (± 2.16)
Infant HC (cm)	31.18 (± 2.62)
Infant HC (*z*‐score)	−5.58 (± 1.65)

*Note:* This table provides a comprehensive summary of the characteristics of the study population, including maternal health indicators, prenatal ultrasound findings, and both immediate and discharge‐related outcomes for offspring. Data are presented as mean ± standard deviation for normally distributed variables, median (interquartile range) for skewed variables, and prevalence (%) with event/total available counts (excluding missing data).

Abbreviations: BE = base excess; CPR = Cerebroplacental Ratio; EFW = Estimated Fetal Weight; GDM = Gestational Diabetes Mellitus; HDP = Hypertensive Disorders of Pregnancy; MCA = Middle Cerebral Artery; MRI = Magnetic Resonance Imaging; pH = potential of hydrogen; PI = Pulsatility Index; RDS = Respiratory Distress Syndrome; UA = Umbilical Artery.

### 3.1 Prenatal Fetal Doppler and Outcomes

**FIGURE 1:**
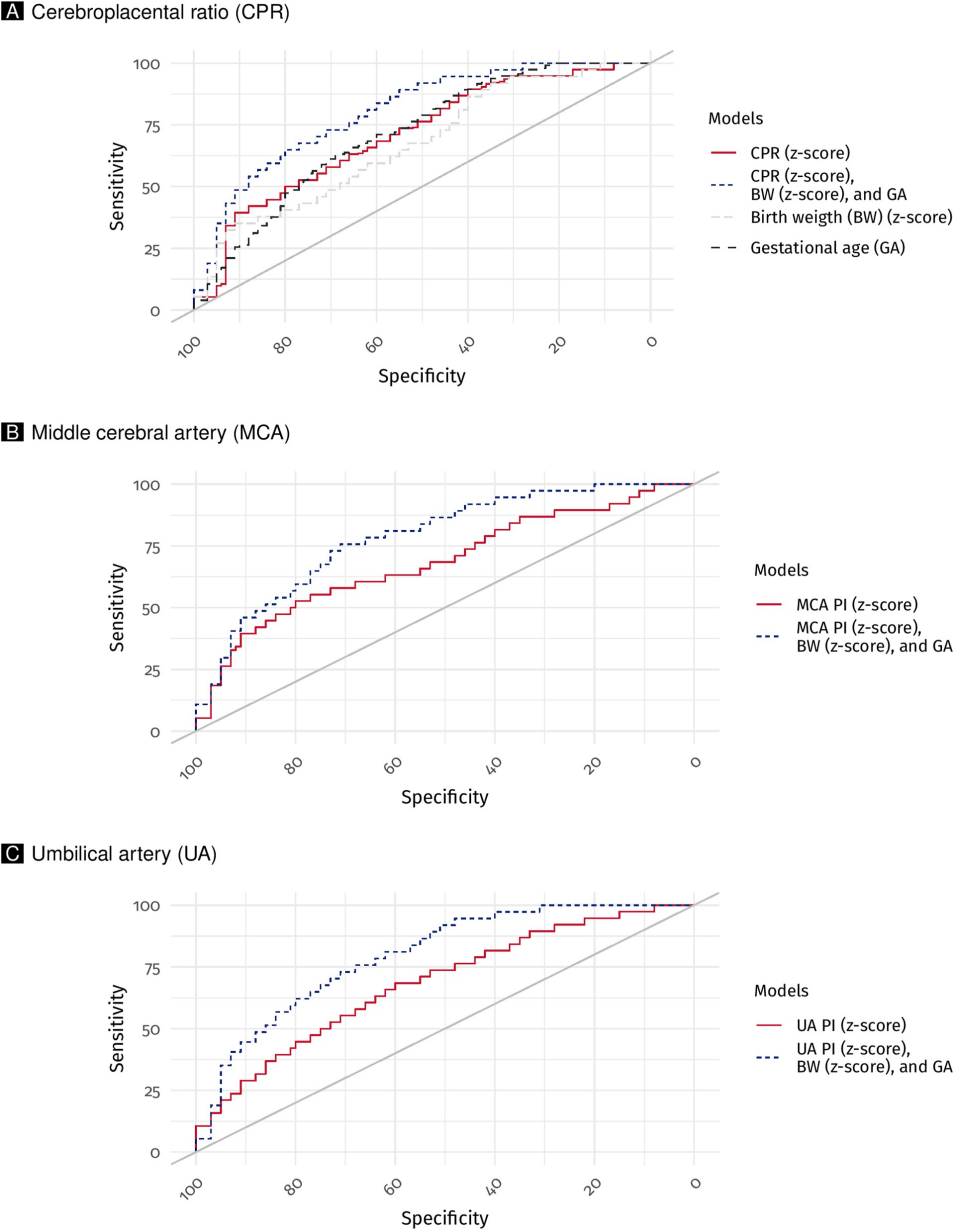
Receiver operating characteristic (ROC) curves used to predict composite neurological outcomes. The diagrams illustrate the ROC curves for different univariate and multivariate logistic regression models to forecast composite neurological outcomes. The models were assessed according to their sensitivity and specificity. Panel A displays ROC curves for the cerebroplacental ratio (CPR) *z*‐score, gestational age (GA), and the combined model of CPR *z*‐score with GA. Panel B displays the ROC curves for the *z*‐score of the middle cerebral artery (MCA) pulsatility index (PI) and the combined model of the MCA PI *z*‐score with gestational age (GA). Panel C displays the ROC curves for the *z*‐score of the umbilical artery (UA) pulsatility index (PI) and the combined model of UA PI *z*‐score with gestational age (GA). The graphs depict the ability of each model to differentiate between infants with and without adverse neurological outcomes. The area under the curve (AUC) quantifies the predictive accuracy of each model.

Univariate and multivariate logistic regression analyses revealed that maternal age and GDM were not significant predictors of composite neurological outcomes (Table 2). The MCA *z*‐score (OR 0.51, 95% CI 0.32–0.82, *p* < 0.05) and UA *z*‐score (OR 1.36, 95% CI 1.1–1.68, *p* < 0.05) were significant predictors in univariate analysis and remained significant in multivariate analysis adjusted for gestational age (MCA *z*‐score OR 0.53, 95% CI 0.32–0.87, *p* < 0.05; UA *z*‐score OR 1.33, 95% CI 1.07–1.66, *p* < 0.05) (Table 2). CPR *z*‐score and neonatal weight *z*‐score were both significant predictors in univariate and multivariate analyses (Table 2 and Figure 1 Panel A).

**TABLE 2:** Logistic regression analyses, both univariable and multivariable, were conducted for the composite neurological and respiratory outcomes.

	OR (CI.95)	*p*	OR (CI.95) (*)	*p*(*)
Neurological composite outcome				
Mother age (years)	1.01 (0.93–1.09)	0.852	1.00 (0.91–1.1)	0.946
HDP	1.47 (0.61–3.55)	0.395	1.12 (0.41–3.07)	0.819
GDM	0.18 (0.02–1.53)	0.115	0.17 (0.02–1.62)	0.123
MCA PI (*z*‐score)	0.51 (0.32–0.82)	< 0.05	0.59 (0.35–0.99)	< 0.05
UA PI (*z*‐score)	1.36 (1.10–1.68)	< 0.05	1.25 (0.99–1.57)	0.057
CPR (*z*‐score)	0.61 (0.44–0.83)	< 0.05	0.67 (0.48–0.95)	< 0.05
Newborn female sex	0.43 (0.18–1.05)	0.064	0.63 (0.23–1.72)	0.370
Birth weight (*z*‐score)	0.47 (0.27–0.80)	< 0.05	0.60 (0.32–1.14)	0.119
Birth length (*z*‐score)	1.14 (0.79–1.65)	0.483	1.51 (0.92–2.49)	0.106
Birth HC (*z*‐score)	0.75 (0.48–1.18)	0.211	0.92 (0.46–1.82)	0.809
Apgar score (1st min)	0.89 (0.69–1.13)	0.335	1.16 (0.83–1.6)	0.383
Apgar score (5th min)	0.80 (0.54–1.17)	0.251	1.2 (0.73–1.96)	0.475
Apgar score (10th min)	1.23 (0.67–2.26)	0.506	1.97 (0.84–4.64)	0.120
Cord blood pH	0.74 (0–191.28)	0.917	1.26 (0–497.75)	0.940
Cord blood BE	0.82 (0.61–1.11)	0.208	0.84 (0.59–1.19)	0.323
Gestational age (days)	0.47 (0.27–0.80)	< 0.05	0.75 (0.61–0.93)	< 0.05
Respiratory composite outcome				
Mother age (years)	0.99 (0.89–1.09)	0.785	0.94 (0.84–1.06)	0.301
HDP	6.03 (1.27–28.61)	< 0.05	3.97 (0.77–20.48)	0.099
GDM	1.48 (0.16–13.2)	0.728	2.16 (0.22–21.66)	0.513
MCA PI (*z*‐score)	0.82 (0.51–1.32)	0.415	0.93 (0.53–1.63)	0.802
UA PI (*z*‐score)	1.12 (0.88–1.42)	0.363	1.07 (0.81–1.4)	0.647
CPR (*z*‐score)	0.9 (0.66–1.23)	0.507	0.98 (0.68–1.41)	0.907
Newborn female sex	0.75 (0.25–2.26)	0.616	0.87 (0.25–3.03)	0.827
Birth weight (*z*‐score)	0.76 (0.41–1.42)	0.395	0.65 (0.26–1.59)	0.342
Birth length (*z*‐score)	1.50 (0.89–2.52)	0.126	1.15 (0.68–1.94)	0.595
Birth HC (*z*‐score)	1.21 (0.69–2.13)	0.500	1.15 (0.61–2.15)	0.668
Apgar score (1st min)	0.7 (0.47–1.05)	0.086	0.81 (0.54–1.23)	0.331
Apgar score (5th min)	0.35 (0.15–0.81)	< 0.05	0.45 (0.19–1.05)	0.064
Apgar score (10th min)	0.55 (0.21–1.43)	0.220	0.54 (0.23–1.31)	0.173
Cord blood pH	0.03 (0–56.56)	0.350	0.09 (0–1744.56)	0.630
Cord blood BE	0.88 (0.61–1.26)	0.478	0.73 (0.45–1.19)	0.205
Gestational age (days)	0.58 (0.42–0.80)	< 0.05	0.56 (0.38–0.82)	< 0.05

*Note:* Results are expressed as odds ratios (ORs) accompanied by 95% confidence intervals (CI.95) and p‐values. Multivariable models (*) were adjusted for gestational age at delivery and birthweight z‐score.

Abbreviations: BE = base excess; CPR = Cerebroplacental Ratio; HDP = GDM = Gestational Diabetes Mellitus; Hypertensive Disorders of Pregnancy; MCA = Middle Cerebral Artery; pH = potential of hydrogen; PI = Pulsatility Index; UA = Umbilical Artery.

The highest predictive factors for neurological outcomes were gestational age and CPR *z*‐score, with AUC values of 71.52% and 71.05%, respectively (Table 3 and Figure 1 Panel A). The multivariate model had an AUC of 80.30%, and significant differences were observed between the univariate and multivariate models (Table 3 and Figure 1 Panel A). Figure 1 (Panels B and C) shows how well MCA and UA PI *z*‐scores predict composite neurological outcomes. For respiratory outcomes, HDP was significant in univariate analysis but not in multivariate analysis (Table 2). Other Doppler measurements were not significant predictors in either the univariate or multivariate analyses (Table 2).

**TABLE 3:** Area under the curve (AUC) values of univariate and multivariate logistic regression models for predicting the composite neurological outcome.

Models	AUC (CI.95)	Gestational age (GA)	Birth weight (BW) (*z*‐score)	MCA PI (*z*‐score)	MCA PI (z‐score), BW (*z*‐score), and GA	CPR (*z*‐score)	CPR (*z*‐score), BW (*z*‐score), and GA	UA PI (*z*‐score)
Gestational age (GA)	71.52% (60.56%–82.48%)							
Birth weight (BW) (*z*‐score)	67.33% (55.64%–79.02%)	0.670						
MCA PI (*z*‐score)	69.12% (57.48%–80.77%)	0.771	0.830					
MCA PI (*z*‐score), BW (*z*‐score), and GA	78.86% (69.06%–88.65%)	0.081	< 0.05	0.057				
CPR (*z*‐score)	71.05% (59.87%–82.23%)	0.954	0.632	0.656	0.126			
CPR (*z*‐score), BW (*z*‐score), and GA	80.30% (70.88%–89.73%)	0.052	< 0.05	< 0.05	0.509	< 0.05		
UA PI (*z*‐score)	68.77% (57.32%–80.23%)	0.730	0.924	0.955	0.087	0.368	< 0.05	
UA PI (*z*‐score), BW (*z*‐score), and GA	79.94% (70.47%–89.41%)	< 0.05	< 0.05	0.091	0.715	0.071	0.822	< 0.05

*Note:* The *p*‐values in the table indicate the differences between the models using DeLong tests. This table displays the Area Under the Curve (AUC) values for different univariate and multivariate logistic regression models that were employed. The AUC values are accompanied by their corresponding 95% confidence intervals (CI).

Abbreviations: AUC = Area Under the Curve; BW = Birth Weight; CI = Confidence Interval; CPR = Cerebroplacental Ratio; GA = Gestational Age; MCA = Middle Cerebral Artery; PI = Pulsatility Index; UA = Umbilical Artery.

**FIGURE 2:**
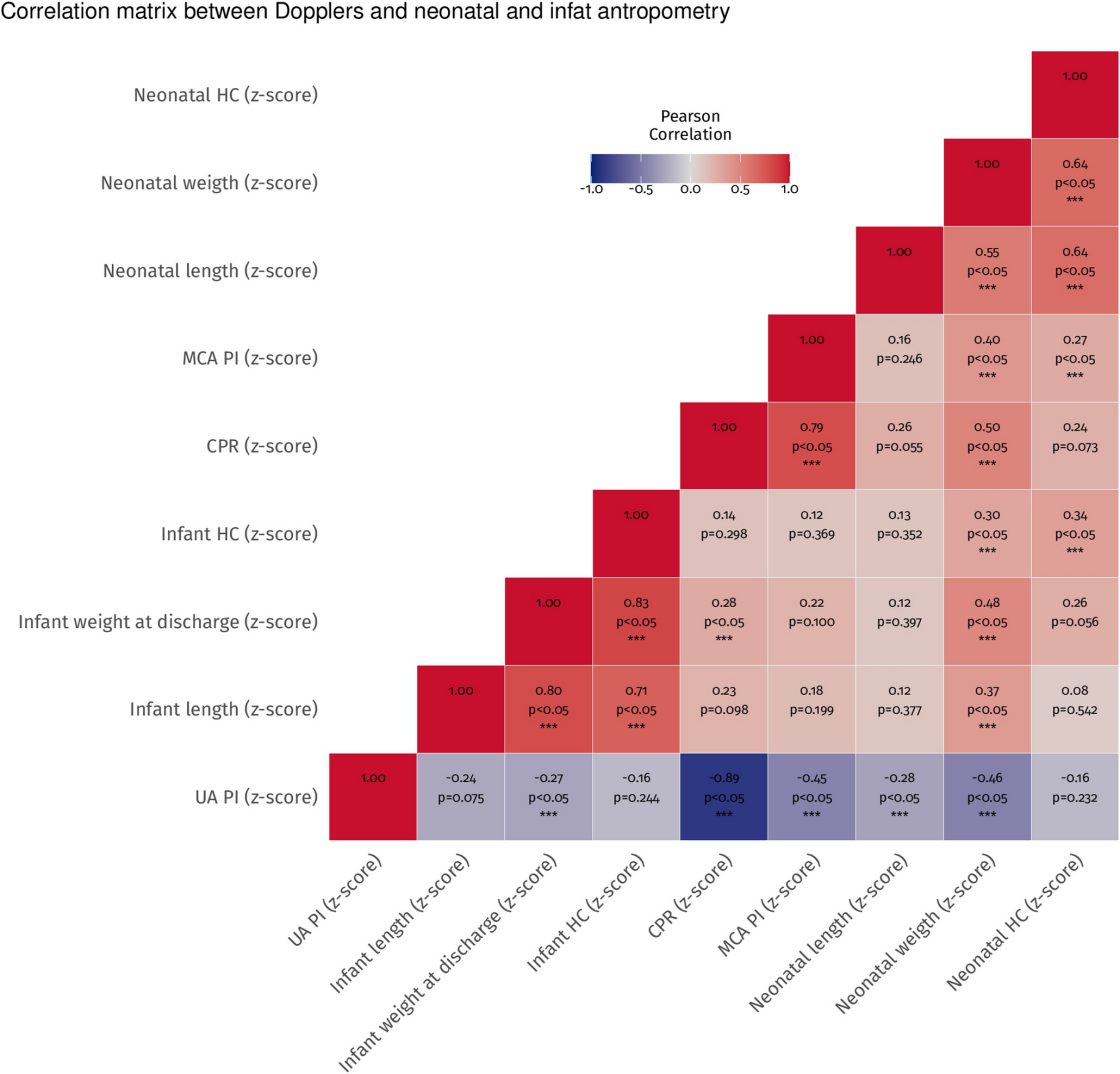
Correlation matrix between Doppler measurements and neonatal and infant anthropometry. This figure illustrates a correlation matrix that shows the Pearson correlation coefficients between different Doppler measurements and neonatal and infant anthropometric outcomes. The matrix comprises the following variables: umbilical artery (UA) pulsatility index (PI) *z*‐score, cerebroplacental ratio (CPR) *z*‐score, middle cerebral artery (MCA) PI *z*‐score, neonatal length *z*‐score (at birth), neonatal weight *z*‐score (at birth), neonatal head circumference (HC) *z*‐score (at birth), infant length *z*‐score (at discharge), infant weight *z*‐score (at discharge), and infant HC *z*‐score (at discharge). The coefficients are accompanied by *p*‐values that indicate the significance of the correlations. Significant correlations are marked by asterisks (*** for *p* < 0.05). The *p*‐values were adjusted for the false discovery rate. The matrix comprehensively explains the correlation between prenatal Doppler measurements and different neonatal and infant growth parameters.

Correlation analysis revealed a significant correlation (*p* < 0.05) between neonatal HC and MCA PI *z*‐score (Figure 2). Also, in univariate linear regression, the correlation was significant but not after adjusting for gestational age and birth weight *z*‐score (coefficient 0.090, 95% CI –0.067/0.248, *p* = 0.256). None of the fetal Doppler measurements was associated with the infant's HC *z*‐score upon discharge. Figure 2 presents the correlation matrix, which reveals several other significant associations. There were strong intercorrelations among neonatal anthropometric *z*‐scores for birthweight, length, and HC. The UA PI *z*‐score exhibited an inverse correlation with the neonatal weight *z*‐score (rho = −0.46, *p* < 0.05), indicating a significant relationship between elevated umbilical artery resistance and compromised fetal growth.

## 4 Discussion

The study's main findings show that both MCA Doppler and CPR are significant predictors of composite neurological outcomes in fetuses with FGR. The MCA *z*‐score and CPR *z*‐score were found to be reliable indicators in both univariate and multivariate logistic regression analyses, even after controlling for gestational age. Furthermore, while HDP was a significant predictor of respiratory outcomes in univariate analysis, it lost its significance in multivariate models. Notably, neonatal HC showed a significant correlation with MCA PI and CPR *z*‐scores. These findings highlight the value of MCA Doppler and CPR in improving the prediction of adverse neurological outcomes.

The study's findings highlight a good predictive value of MCA Doppler and CPR measurements for neurological outcomes in FGR fetuses. This finding is consistent with previous research highlighting the importance of Doppler assessments in prenatal care. Prior research, such as that conducted by Botsis et al. (2006) and Thomas et al. (2018), has demonstrated the utility of Doppler velocimetry in monitoring fetal well‐being, particularly in cases of FGR [27, 28]. These studies have found that abnormal Doppler readings, including those of the MCA and CPR, are associated with poor perinatal outcomes.

Our findings are consistent with Botsis et al. (2006), who found that decreased MCA resistance, combined with increased UA resistance, is a critical marker of fetal hypoxia and can guide clinical decisions about delivery timing to reduce perinatal risks [27]. Similarly, Mendoza et al. (2021) found that improved placental perfusion and angiogenic profiles, as determined by Doppler assessments, are associated with better fetal outcomes in severe FGR cases [29].

Furthermore, our study identified a significant correlation between neonatal HC, MCA PI, and CPR *z*‐scores. This result is consistent with findings by Morgan et al. (2014), who also reported a similar correlation and underscored the significance of prenatal Doppler assessments in predicting long‐term neurodevelopmental outcomes in preterm infants [5]. Additionally, Bartholomeusz et al. (2002) validated HC as a surrogate measure of brain volume, further emphasizing the importance of HC measurements in early neurological assessments [30]. The consistent findings across these studies highlight the clinical implications of incorporating MCA and CPR Doppler measurements into routine prenatal assessments to enhance early detection and intervention strategies for fetuses at risk of adverse neurological outcomes. However, our study did not find a correlation between fetal Doppler and infant HC, likely due to the amount of missing data. Therefore, more evidence is needed to draw definitive conclusions.

A link between prenatal Doppler measurements and postnatal outcomes has been found in several studies. Previous research, for example, has examined the impact of timely detection and intervention in small‐for‐gestational‐age fetuses, emphasizing the importance of Doppler assessments in improving neonatal outcomes [7, 20]. Similarly, Zhang‐Rutledge et al. (2018) highlighted the importance of advanced diagnostic tools, such as Doppler ultrasound, for tracking fetal growth and predicting complications [31].

Our study adds to the growing body of evidence by thoroughly analyzing the predictive accuracy of these Doppler measurements using univariate and multivariate logistic regression models. Even after adjusting for gestational age, the MCA *z*‐score and CPR *z*‐score have a significant predictive value, demonstrating their robustness as tools. These findings emphasize the need for ongoing research and validation of Doppler assessments to improve clinical outcomes in high‐risk pregnancies [1]. Senra et al. (2020) on the effect of FGR on renal development, and Franz et al. (2009) on growth trajectories in very‐low‐birth‐weight infants highlight the critical need for comprehensive prenatal and postnatal care strategies that include Doppler assessments [32, 33].

Our findings are consistent with those of the TRUFFLE study, which highlighted the importance of detailed Doppler monitoring in improving perinatal outcomes for growth‐restricted fetuses [34, 35]. Stampalija et al. (2017), in particular, highlighted the association between MCA Doppler parameters and neonatal survival without severe neurological morbidity, corroborating these measures' predictive value [36]. Cord blood pH did not demonstrate discriminatory value for composite neurological outcomes, exhibiting unstable logistic regression estimates and extremely wide confidence intervals resulting from near‐complete overlap between outcome groups. This finding is not unexpected, as cord blood pH primarily indicates intrapartum events rather than the total fetal hemodynamic burden accumulated in the preceding weeks. In contrast, prenatal Doppler parameters provide longitudinal data on cerebral blood flow redistribution and placental resistance, offering a prognostic value that exceeds acute perinatal markers and demonstrating consistent and significant performance in both univariate and multivariable analyses.

The study's strengths include the setting in a high‐risk clinic and the careful selection of a well‐defined cohort, both of which improve the reliability and relevance of the results. However, there are several limitations. It should be noted that the relatively high composite neurological outcome rate (45.78%) in our cohort was largely driven by pathological findings on neonatal brain ultrasound (39.76%), which may include imaging abnormalities of variable clinical significance that do not necessarily correspond to long‐term neurological impairment. Severe structural injuries, such as intraventricular hemorrhage and periventricular leukomalacia, were observed in only 4.94% and 0% of cases, respectively. The composite outcome, therefore, captures a spectrum of neurological risk rather than exclusively major morbidity, and findings should be interpreted within this context. The retrospective design and reliance on clinical chart reviews may introduce biases due to insufficient or inconsistent data collection. The exclusion of cases without ultrasound data within 1 week before birth may limit generalizability. Furthermore, the small sample size may limit statistical power and precision, especially in subgroup analyses. Focusing on a single center may fail to capture differences in clinical practices and patient populations across settings.

The results of this study must be interpreted in the context of a highly selected high‐risk obstetric population. This cohort exhibited a significant prevalence of HDP and GDM, alongside a sole reliance on cesarean birth. The latter is a significant factor for generalizability, as it represents the management protocol of our high‐risk unit during the study period and may not accurately reflect centers where vaginal birth is more frequently pursued in pregnancies complicated by FGR. In these contexts, the clinical characteristics of FGR patients and the associations between prenatal Doppler parameters and neonatal outcomes may vary. Consequently, it is essential to exercise caution when generalizing these findings to wider FGR populations or to obstetric environments characterized by varying birth practices, risk profiles, and institutional protocols.

The study's findings have prominent implications for clinicians, policymakers, and healthcare providers. The predictive value of MCA Doppler and CPR measurements underscores the need to incorporate these tests into routine prenatal care for fetuses at risk of FGR. Early detection of adverse neurological outcomes allows for timely and targeted interventions, potentially improving long‐term developmental outcomes. Policymakers and healthcare providers should take these findings into account when developing guidelines for managing high‐risk pregnancies and investing in Doppler ultrasound training and resources to improve prenatal care quality.

Despite the valuable insights gained, many questions remain unanswered. Future research should investigate the long‐term outcomes of infants identified as at risk using MCA Doppler and CPR measurements, and validate these findings in larger, multicenter studies with diverse populations. Furthermore, investigating the integration of additional predictive markers, such as biochemical and genetic factors, can help develop more comprehensive models to predict neurological outcomes in FGR. Understanding the underlying mechanisms that link Doppler measurements to neurological outcomes may provide additional insights into FGR's pathophysiology and inform novel therapeutic strategies.

In conclusion, this study highlights the excellent predictive value of multivariate models, including MCA Doppler and CPR measurements, in assessing neurological outcomes in FGR fetuses. Our findings show that MCA Doppler and CPR *z*‐scores accurately predict adverse neurological outcomes, even after accounting for gestational age. These findings demonstrate the clinical utility of incorporating Doppler assessments into routine prenatal care for high‐risk pregnancies, allowing early detection and intervention to improve long‐term developmental outcomes.

## Funding

The authors declare that no funds, grants, or other support were received during the preparation of this manuscript.

## Ethics Statement

The local ethical committee approved the data analysis and publication. All women signed informed consent forms to allow their clinical data to be anonymously used in scientific publications. This investigation was carried out following the instructions of national health authorities and good clinical practice procedures.

## Consent

The authors have nothing to report.

## Conflicts of Interest

The authors declare no conflicts of interest.

## Data Availability

The data that support the findings of this study are available. However, restrictions apply to the availability of these data, which were used under license for the current study and are not publicly available. Data are, however, available from the authors upon reasonable request and with permission from the Internal Review Board.
